# HR-LC-ESI-Orbitrap-MS-Based Metabolic Profiling Coupled with Chemometrics for the Discrimination of Different *Echinops spinosus* Organs and Evaluation of Their Antioxidant Activity

**DOI:** 10.3390/antiox11030453

**Published:** 2022-02-24

**Authors:** Amel Bouzabata, Paola Montoro, Katarzyna Angelika Gil, Sonia Piacente, Fadia S. Youssef, Nawal M. Al Musayeib, Geoffrey A. Cordell, Mohamed L. Ashour, Carlo Ignazio Giovanni Tuberoso

**Affiliations:** 1 Department of Pharmacy, Faculty of Medicine, Zaafrania Street BP 205, Annaba 23000, Algeria; 2 Department of Pharmacy, University of Salerno, Via Giovanni Paolo II, 132, 84084 Fisciano, SA, Italy; pmontoro@unisa.it (P.M.); piacente@unisa.it (S.P.); 3 Department of Life and Environmental Sciences, University of Cagliari, University Campus, S.P. Monserrato-Sestu km 0.700, 09042 Monserrato, CA, Italy; kasiagil8a@gmail.com; 4 Department of Pharmacognosy, Faculty of Pharmacy, Ain-Shams University, Abbasia, Cairo 11566, Egypt; fadiayoussef@pharma.asu.edu.eg; 5 Department of Pharmacognosy, College of Pharmacy, King Saud University, Riyadh 11495, Saudi Arabia; nalmusayeib@ksu.edu.sa; 6 Natural Products Inc., Evanston, IL 60202, USA; pharmacog@gmail.com; 7 Department of Pharmaceutics, College of Pharmacy, University of Florida, Gainesville, FL 32610, USA

**Keywords:** antioxidant activity, chemometrics, *Echinops*, phenolics, HPLC-DAD, ADME/TOPAKT

## Abstract

This study aimed to assess and correlate the phenolic content and the antioxidant activity of the methanol extracts of the stems, roots, flowers, and leaves of *Echinops spinosus* L. from north-eastern Algeria. Qualitative analysis was performed by high-resolution mass spectrometry (HR) LC-ESI-Orbitrap-MS and (HR) LC-ESI-Orbitrap-MS/MS). Forty-five compounds were identified in the methanol extracts; some are described for the first time in *E. spinosus.* Targeted phenolic compounds were quantified by HPLC-DAD and it was shown that caffeoyl quinic derivatives were the most abundant compounds. Chemometric analysis was performed using principal component analysis (PCA) and hierarchical cluster analysis (HCA) based on the qualitative and quantitative LC data. The score plot discriminates different *Echinopsis spinosus* organs into three distinct clusters, with the stems and flowers allocated in the same cluster, reflecting their resemblance in their secondary metabolites. The antioxidant activities of the methanol extracts were assessed using cupric reducing antioxidant capacity (CUPRAC), ferric reducing antioxidant assay (FRAP), diphenyl picryl hydrazyl radical-scavenging capacity assay (DPPH**^●^**), and 2,2′-azinobis-(3-ethylbenzothiazoline-6-sulfonic acid (ABTS**^●^**^+^). The root extract exhibited the highest antioxidant activity, evidenced by 3.26 and 1.61 mmol Fe^2+^/g dried residue for CUPRAC and FRAP, respectively, and great free radical-scavenging activities estimated by 0.53 and 0.82 mmol TEAC/g dried residue for DPPH**^●^** and ABTS**^●^**^+^, respectively. The methanol extract of the roots demonstrated a significant level of total phenolics (TP: 125.16 mg GAE/g dried residue) and flavonoids (TFI: 25.40 QE/g dried residue TFII: 140 CE/g dried residue). Molecular docking revealed that tricaffeoyl-altraric acid and dicaffeoyl-altraric acid exhibited the best fit within the active sites of NADPH oxidase (NO) and myeloperoxidase (MP). From ADME/TOPAKT analyses, it can be concluded that tricaffeoyl-altraric acid and dicaffeoyl-altraric acid also revealed reasonable pharmacokinetic and pharmacodynamic characteristics with a significant safety profile.

## 1. Introduction

Oxidative stress can be defined as a condition in which the oxidative forces override the antioxidant mechanisms within the body owing to the absence of balance among them. It is implicated in the pathogenesis of a plethora of widely spread ailments that are closely related to lifestyle, comprising hypertension, atherosclerosis, ischemic heart diseases, diabetes mellitus, and cancer [1,2]. Free radicals are considered naturally occurring reactive compounds inside the human body. They can elicit both beneficial and harmful effects, with the former observed in the immune system and the latter evident in proteins, lipids, and DNA [3]. Thus, the antioxidant system that constitutes complex protection is highly demanded by living organisms to oppose and abolish these hazardous effects. Plant-derived polyphenolic compounds have long been used as a promising source of antioxidants that can boost the antioxidant system and counteract oxidative stress [2,4].

The genus *Echinops* L. (Asteraceae) comprises about 130 species, many of which are used in traditional medicine, mainly in Africa and Asia [5]. *E. spinosus* (synonyms: *E. spinosissimus* Turra) is distributed in North Africa, the Mediterranean basin, and temperate regions towards Central Asia [6]. *E. spinosus* is widely used in traditional medicine to treat inflammation-related diseases [7]. The internal part of the inflorescence is used for kidney ailments [8], in post-partum care [9], and as a hypoglycemic plant for treating diabetes mellitus [10]. 

In Algeria, the roots and fruits of *E. spinosus* subsp. *bovei* are used as an abortifacient and for treating labor pains and neuralgia [11], and as a spice in Morocco and Cameroon [12,13]. Meanwhile, a decoction of the roots in either water or olive oil is given to help pregnant women in delivery via stimulation of uterine contractions. It is also used for stomach pain, indigestion, and lack of appetite; for diabetes as a diuretic or depurative; and to cure liver diseases [14]. These activities are strongly correlated with the abundance of secondary metabolites belonging to diverse classes such as alkaloids, sesquiterpenes, flavonoids, and polyphenolic compounds in different organs of *E. spinosus* [15]. 

By evaluating the current literature, it was clear that the information on the metabolites of *E. spinosus* is fragmentary due to the differences in both the solvents and plant parts used for extraction. Therefore, a more detailed investigation of the methanol extracts prepared from the stems, roots, flowers, and leaves of *E. spinosus* was performed. Qualitative analysis was performed by high-resolution mass spectrometry ((HR) LC-ESI-Orbitrap-MS and (HR) LC-ESI-Orbitrap-MS/MS) and targeted phenolic compounds were quantified using HPLC-DAD. A chemometric analysis represented by principal component analysis (PCA) and hierarchical cluster analysis (HCA) was performed, which relied upon the collected LC data, aiming to classify the measured samples into discriminant clusters according to the quantity and quality of polyphenolic compounds that in turn reflected on its biological activity. Besides, the antioxidant activities of the methanol extracts were assessed using different antioxidant tests, namely, the cupric reducing antioxidant capacity (CUPRAC), ferric reducing antioxidant assay (FRAP), diphenyl picryl hydrazyl radical-scavenging capacity assay (DPPH**^●^**), and 2,2′-azinobis-(3-ethylbenzothiazoline-6-sulfonic acid (ABTS**^●^**^+^). Correlations were done based on the total phenol and the total flavonoid contents determined by spectrophotometric assays. Furthermore, molecular docking of the major polyphenolic compounds identified from different organs of the *E. spinosus* methanol extract was performed within the active sites of two enzymes that are responsible for the production of (ROS), which are NADPH oxidase (NO) and myeloperoxidase (MP). Meanwhile, the pharmacodynamic, pharmacokinetic, and toxicity properties of these compounds were exposed to ADMET evaluation (absorption, distribution, metabolism, excretion, and toxicity), as well as to toxicity prediction (TOPKAT) studies.

## 2. Materials and Methods

### 2.1. Chemicals and Reagents

All the solvents and chemicals used in this study were analytical grade. Methanol, ethanol, acetonitrile LC-MS grade, formic acid LC-MS grade, 85% phosphoric acid, and water LC-MS grade were purchased from Merck^®^ (Darmstadt, Germany). Gallic acid, quinic acid, caffeic acid, ferulic acid, protocatechuic acid, neochlorogenic acid (3-*O*-caffeoylquinic acid), chlorogenic acid (5-*O*-caffeoylquinic acid), cynarin (1,3-dicaffeoylquinic acid), isochlorogenic acid, kaempferol-3-*O*-glucoside, quercetin-3-*O*-glucoside, ferrous sulfate, 1,1-diphenyl-2-picrylhydrazyl radical (DPPH**^●^**), (±)-6-hydroxy-2,5,7,8-tetramethylchroman-2-carboxylic acid (Trolox), 2,4,6-tris(2-pyridyl) -1,3,5-triazine (TPTZ), 2,2′-azino-bis(3-ethylbenzothiazoline)-6-sulfonic acid (ABTS), Folin–Ciocalteu reagent, sodium carbonate, ferric chloride, AlCl_3_, NaNO_2_, and CuSO_4_∙5H_2_O were purchased from Merck^®^, Sigma-Aldrich^®^, or Fluka™ (Milan, Italy). Flavonoid standards of apigenin, apigenin-7-*O*-glucoside, hesperidin, hesperetin, luteolin-7-*O*-glucoside, naringin, and naringenin were purchased from Extrasynthese (Genay, France). Ultrapure water (18 MΩ·cm) was obtained with a Milli-Q Advantage A10 System apparatus (Millipore, Milan, Italy).

### 2.2. Plant Materials

Samples of *Echinops spinosus* subsp. *bovei* (Boiss) Murb were wild-collected in 2017 from the El Tarf district situated in northeastern Algeria. Voucher specimens were deposited in the herbarium of the Conservatory and Botanical Garden, Geneva, Switzerland, under reference number G00403753. Identification of the species was carried out by Dr. G. Debelaire by correlating the morphological characters with those described in the literature and identified as *E. bovei* Maire [16]. Samples of *E. spinosus* were divided into four parts: stems (S), roots (R), flowers (F), and leaves (L). The different plant parts were cleaned, dried in the shade, and crushed into powder, as traditionally used. 

### 2.3. Preparation of the Aqueous Methanol Extracts

Extracts were prepared by the addition of 20 mL of 80% methanol to 2 g of dried powdered plant material. Extraction was performed using ultrasonification for 30 min at 15 °C, followed by centrifugation for 15 min at 10 °C using 4000 rpm. The liquid phase was separated from the plant material and filtered using 0.45 μm CA w/GMF Whatman. The extraction procedure was repeated on the marc and the liquid phases were combined (final dilution 1:20, *w*/*v*) with methanol. Solutions were appropriately diluted in the range of 1:10–1:100 *v*/*v* for the cupric reducing antioxidant capacity (CUPRAC), ferric reducing antioxidant assay (FRAP), diphenyl picryl hydrazyl radical-scavenging capacity assay (DPPH·), 2,2′-azinobis-(3-ethylbenzothiazoline-6-sulfonic acid (ABTS**^●^**^+^), total phenolic (TP), and total flavonoids (TF) assays. For LC-MS and LC-DAD analysis, methanol extracts were dried under N_2_, and a sample (1 mg) was dissolved in MeOH:H_2_O (1:1) to a final concentration of 1 mg/mL. The dry residue of the plant extracts was evaluated in triplicate by drying the solution (500 µL) for 5 h in a thermostatic oven at 105 ± 1 °C to constant weight.

### 2.4. Qualitative Investigation of E. spinosus Using LC-ESI-Orbitrap-MS and LC-ESI-Orbitrap-MS/MS and LC-DAD Analysis

For the qualitative investigation of the methanol extracts of different *E. spinosus* organs, LC-ESI-(LIT) MS and LC-ESI-(LIT) MS/MS techniques were performed. Preliminarily, the electrospray ionization (ESI) source of a Thermo Scientific LTQ-Orbitrap XL (Thermo Scientific, Dreieich, Germany) mass spectrometer was tuned in the negative ion mode with a standard solution (1 µg/mL) of kaempferol-3-*O*-glucoside and injected at a flow rate of 5 μL/min using a syringe pump. Calibration of the Orbitrap analyzer used the standard LTQ calibration mixture composed of caffeine and the peptide MRFA (from the manufacturer Thermo Scientific, Dreieich, Germany) dissolved in 50:50 *v*/*v*% water/acetonitrile solution. The resolution for the Orbitrap mass analyzer was set at 30,000. Spectra were acquired by full-range acquisition total ion current (TIC) covering a range of *m/z* 180–1600. When working in LC-MS, instrument control, data acquisition, and data processing were performed with Xcalibur 2.0 software (Thermo Fisher Scientific, Bremen, Germany). LC/ESI/LIT Orbitrap MS experiments were achieved using a Thermo Fisher Scientific liquid chromatography system comprised of a quaternary Accela 600 pump and an Accela autosampler, connected to a linear Trap-Orbitrap hybrid mass spectrometer (LTQ-Orbitrap XL, Thermo Fisher Scientific) with electrospray ionization (ESI). LC-ESI-Orbitrap-MS analyses were performed using a Phenomenex Luna C18 (150 mm × 2.1 mm, particle size 5 µm) column, eluted with water containing 0.1% formic acid (solvent A) and acetonitrile containing 0.1% formic acid (solvent B). A linear gradient program at a flow rate of 0.200 mL/min was adopted employing the following protocol: 0–35 min, from 5 to 95% (B), and 35–40 min, returning to 5% and back to 5% (B) for an additional 5 min. A total of 10 µL of a solution (1 mg/mL) of the extract in water was injected. The ESI source and MS parameters were as follows: capillary voltage −12 V; tube lens voltage −121.47; capillary temperature 280 °C; sheath and auxiliary gas flow (N_2_) 15 and 5, respectively; sweep gas 0; spray voltage 5. MS spectra were acquired by full-range acquisition covering *m/z* 180–1400. For fragmentation studies, a data-dependent scan experiment was performed, selecting precursor ions corresponding to the two most intensive peaks in the LC-MS analysis.

### 2.5. Quantitative Determination of E. spinosus Major Phenolic Compounds Using HPLC-DAD

Quantitative analysis on targeted phenolic compounds was carried out using a modified HPLC-DAD method, as previously described by Deiana et al. [17]. Polar compounds were identified and determined using an Agilent 1260 Infinity II HPLC system (Varian, Leinì, TO, Italy) fitted with a pump module G7111A, an autosampler module G7129A, and an Agilent G4212B photodiode array detector (Agilent Technologies, Cernusco sul Naviglio, MI, Italy). Separation was obtained with a Kinetex PFP C18 column (150 × 4.60 mm, 5 μm, Phenomenex, Casalecchio di Reno, Bologna, Italy) using 0.22 M phosphoric acid (solvent A) and acetonitrile (solvent B) as mobile phase at a constant flow rate of 1.0 mL/min. The gradient (*v*/*v*) was generated by decreasing from 100% solvent A to 80% in 20 min, to 70% in 35 min, to 0% in 45 min, and then remaining stable up to 50 min; finally, the gradient reached 100% and was stabilized for 5 min before the subsequent injection. The chromatograms and spectra were elaborated with an OpenLab V. 2.51 data system (Agilent Technologies, Cernusco sul Naviglio, MI, Italy), and flavonoids were detected and measured at 360 nm, ferulic acid derivatives at 313 nm, and all the other metabolites at 280 nm. Stock standard solutions were prepared in methanol, and the working standard solutions were prepared in ultrapure water. The calibration curves for commercial standards were plotted with the method of the external standard, correlating the peak area with the concentration by means of the least-squares method, with a coefficient of determination (r^2^) > 0.998 in the range of 0.4–40 mg/L for all the compounds. Limits of detection and quantification (LOD and LOQ, respectively) were evaluated in agreement with the International Conference on Harmonisation of Technical Requirements for Registration of Pharmaceuticals for Human Use (ICH) guidance note that describes the validation of analytical methods (ICH Topic Q2, 2006).

### 2.6. Discrimination of the Different E. spinosus Organs Using Multivariate Data Analysis

Chemometric analysis represented by principal component analysis (PCA) and hierarchical cluster analysis (HCA) was performed that relied upon the collected LC data. PCA as an unsupervised pattern recognition technique was conducted to provide a clear insight for all observations that were collected from the samples and then they were classified into discriminant classes in accordance with the quantity and quality of major phenolic compounds that undoubtedly influence the antioxidant activity. Furthermore, HCA was performed, adopting the entire linkage approach used for group classification. PCA and HCA were performed using CAMO’s Unscrambler^®^ X 10.4 software (Computer-Aided Modeling, As, Norway) as previously described [18,19]. 

### 2.7. In Vitro Evaluation of the Antioxidant Activity of Different E. spinosus Organs

#### 2.7.1. Cupric Reducing Antioxidant Capacity (CUPRAC) Assay

The cupric ion-reducing antioxidant capacity (CUPRAC) assay is based upon the redox reaction, producing a chromogen of Cu(I)-neocuproine. The reaction was monitored by spectrophotometric measurements where absorbance was measured at 450 nm. A total of 1 mL water, 500 µL copper (II) chloride, 500 µL neocuproine, 500 µL ammonium acetate, and 100 µL methanol (blank), standard, or sample were added to 10 mm polystyrene cuvettes in that order. Quantitative analysis was performed using the external standard method (ferrous sulfate, 0.1–2 mmol), correlating the absorbance (λ 450 nm) with the concentration, and the spectrophotometric readings were carried out on a Varian Cary 50 Scan spectrophotometer (Varian, Leini, Turin, Italy). The results are expressed as millimoles of Fe^2+^ per g of dry extract [20]. 

#### 2.7.2. Ferric Reducing Antioxidant Assay (FRAP Assay) 

Ferric reducing antioxidant assay (FRAP) is based upon the reduction of ferric 2,4,6-tris(2-pyridyl)-1,3,5-triazine (Fe(III)-TPTZ) to blue-colored ferrous complex by antioxidants in the presence of acidic medium. The reduction was monitored by spectrophotometric measurements of absorbance at 593 nm using a Varian Cary 50 spectrophotometer. Two mL of freshly prepared reagent that was composed of 0.3123 g TPTZ and 0.5406 g FeCl_3_.6H_2_O in 100 mL acetate buffer of pH 3.6 were added to 20 µL of the extract solution with a concentration of 1:200 *w*/*v* in 10 mm polystyrene cuvettes. Quantitative analysis was done using the external standard method, employing ferrous sulfate in the range of 0.1–2 mmol, correlating the absorbance at λ = 593 nm with the concentration. The results were expressed as millimoles of Fe^2+^ per g of dry extract [20].

#### 2.7.3. Diphenyl Picryl Hydrazyl Radical-Scavenging Capacity Assay (DPPH**^●^**)

This method is based on the scavenging of DPPH radicals by antioxidants present in the sample. DPPH**^●^** radicals absorbed at 517 nm, and the reaction was monitored by spectrophotometric measurement, where a decrease in absorbance was observed. A total of 50 µL of extract or standard in the concentration of 1:200 *w*/*v* (using methanol as solvent of dilution) was added to 10 mm cuvettes with 2 mL DPPH**^●^** solution (0.04 mmol/L in methanol) with concomitant mixing. The spectrophotometric readings were carried out on a Varian Cary 50 spectrophotometer at 517 nm after 60 min. A calibration curve in the range of 0.02–1.0 mmol/L was prepared for Trolox, and the data are expressed as the Trolox equivalent antioxidant capacity (TEAC mmol/g dried residue) [20].

#### 2.7.4. Free Radical-Scavenging Ability Determination Using a Stable 2,2′-azino-bis(3-ethylbenzothiazoline)-6-sulfonic acid Radical Cation (ABTS**^●^**^+^)

An ABTS radical cation (ABTS**^●^**^+^) was produced by reacting ABTS stock solution with 70 mM potassium persulfate (final concentration), and the mixture was allowed to stand in the dark at room temperature for 12–16 h before use. After this time, 4 mL of the reaction mixture were diluted with water and 0.08 mM ABTS solution was obtained. The preparation absorbance was checked (0.70 ± 0.02) at 734 nm. The ABTS**^●^**^+^ radical absorbs at 734 nm, and the reaction was monitored by spectrophotometric measurement of the decrease in absorbance. A total of 20 µL of the extract or the standard in the concentration of 1:200 *w*/*v* was added to 10 mm cuvettes with 2 mL of ABTS solution (0.08 mmol/L in H_2_O) and mixed. The spectrophotometric readings were carried out on a Varian Cary 50 spectrophotometer at 734 nm immediately after sample preparation. The calibration curve in the range of 0.02–1.0 mmol/L was prepared for Trolox, and the data are expressed as Trolox equivalent antioxidant capacity (TEAC mmol/g dried residue) [20].

### 2.8. Determination of the Total Phenolic Content

The total phenolic content was determined spectrophotometrically using a modified Folin–Ciocalteu method [17]. Briefly, in each volumetric flask, 500 µL of Folin–Ciocalteu phenol reagent were added to 100 µL of 1:200 (*w*/*v*) of the tested samples. After 5 min, 3 mL of 10% Na_2_CO_3_ (*w*/*v*) were added, and the mixture was shaken and diluted with H_2_O to a final volume of 10 mL. After incubation for 90 min at room temperature, the absorbance was read at 725 nm using a 10 mm polystyrene cuvette with a Varian Cary 50 spectrophotometer against a blank. The total phenolic content was expressed as mg/g of gallic acid equivalent (GAE) using a calibration curve derived from freshly prepared gallic acid standard solutions (10–200 mg/g dried residue) [20].

### 2.9. Determination of Total Flavonoid Content

Total flavonoid (TF) content was determined according to the method previously described by Pękal and Pyrzynska [21] with some modifications [17] using two assays. In the first assay that was given the abbreviation TF1, 200 μL of the diluted sample were mixed with 1.5 mL water and added to 500 μL of 2% (*w*/*v*) aqueous AlCl_3_ solution. The mixture was allowed to stand at room temperature for 30 min and absorbance was determined at 425 nm. Meanwhile, in the second assay that was termed TF2, 200 μL of the diluted sample were mixed with 1 mL water and added to 100 μL of 5% (*w*/*v*) aqueous NaNO_2_ solution. After 5 min, 500 μL of 2% (*w*/*v*) of aqueous AlCl_3_ solution were added, then 500 μL of 1 M NaOH were added after 6 min, followed by incubation for 10 min. Then the absorbance was determined at 510 nm. The results for TF are expressed as mg/g dried residue of quercetin (QE) or catechin CE) equivalent for assays 1 and 2, respectively.

### 2.10. Computer-Aided Drug Design Studies

#### 2.10.1. Molecular Docking

Molecular docking was performed for the major polyphenolic compounds identified from different organs of *E. spinosus* methanol extract within the active sites of two enzymes that are responsible for the production of (ROS), which are NADPH oxidase (NO) (PDB ID: 2CDU; 1.80 Å) and myeloperoxidase (MP) (PDB ID: 5WDG; 2.40 Åobtained from the protein data bank (PDB). The docking study was performed using Discovery Studio 4.5 (Accelrys Inc., San Diego, CA, USA) employing the C-Docker protocol as previously described [18,22]. Meanwhile, the binding energies (∆*G*) were calculated in accordance with the following equation [22,23]:

Δ*G*_binding_ = E_complex_ − (E_protein_ + E _ligand_) (1)

where:

Δ*G*_binding_: the ligand–protein interaction binding energy;

E_complex_: the potential energy for the complex of the protein bound with the ligand; 

E_protein_: the potential energy of the protein alone and;

E_ligand_: the potential energy for the ligand alone.

#### 2.10.2. ADME/TOPKAT Prediction

To determine the pharmacodynamic, pharmacokinetic, and toxicity properties of the major polyphenolic compounds identified from different organs of *E. spinosus* methanol extract, they were exposed to ADMET evaluation (absorption, distribution, metabolism, excretion, and toxicity) as well as to toxicity prediction (TOPKAT) employing Discovery Studio 4.5 software (Accelrys Inc., San Diego, CA, USA). Plasma protein-binding prediction (PPB), human intestinal absorption (HIA), blood–brain barrier penetration (BBB), aqueous solubility, cytochrome P450 2D6, and hepatotoxicity level were chosen as the ADMET parameters. However, Ames mutagenicity, dermal and ocular irritation, and carcinogenic effect on male and female rat NPT (National Toxicology Program), as well as chronic LOAEL (lowest observed adverse effect level) and rat oral and inhalational LD50 and aerobic biodegradability, were selected as TOPKAT descriptors [24,25].

### 2.11. Statistical Analyses

All measurements were conducted in triplicate using one-way analysis of variance (ANOVA) followed by Tukey’s test, which was performed to ascertain the possible significant differences between groups using the Graph Pad Prism 5 software (Graph Pad software, San Diego, CA, USA). Correlation analysis was performed and the evaluation of statistical significance of observed differences was performed using Pearson coefficients of correlation.

## 3. Results and Discussion

The dry residues obtained after extraction of the dried plants showed different yields depending on the botanical part extracted. The lower extraction yields were observed from the root and stem parts estimated by 0.05 ± 0.01 and 0.10 ± 0.01 g/g dried plants, respectively, whereas the flowers and leaves showed higher yields of 0.15 ± 0.01 and 0.20 ± 0.01 g/g dried plants, respectively. These differences can be explained by the virtue of the difference in structure between different tissues, which might hinder extraction, and/or the amount of polar compounds and their solubilization rate in alcohol [26]. 

### 3.1. LC-ESI-Orbitrap-MS and (HR) LC-ESI-Orbitrap-MS/MS and LC-DAD Analysis of E. spinosus Extracts

**Figure 1:**
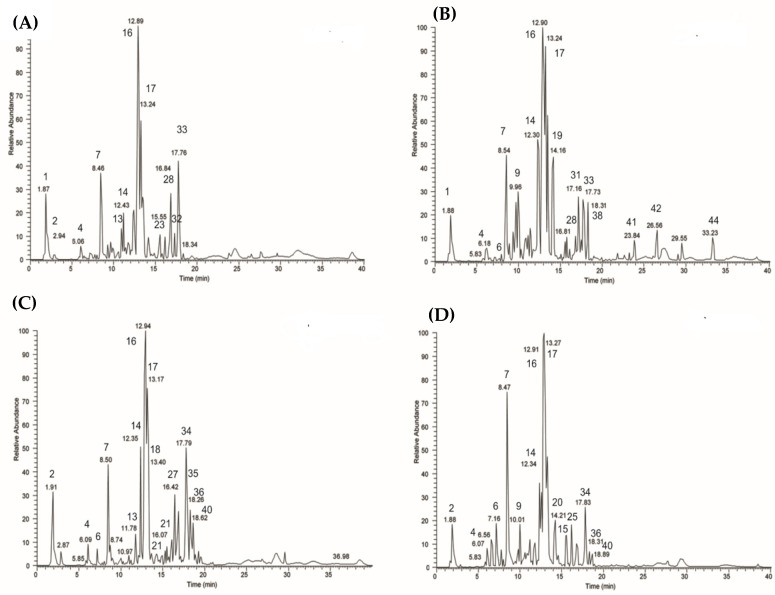
LC-ESI-Orbitrap-MS of extract obtained from the stems (**A**), roots (**B**), flowers (**C**), and leaves (**D**) of *Echinopsis spinosus* L.

The main interest in performing the metabolic profiling of *E. spinosum* relied on the limited information about the chemical content of the different plant parts, and this concomitantly resulted in the performance of qualitative liquid chromatography coupled with the high-resolution mass spectrometry (HPLC-MS) method, aiming to identify the polar metabolites occurring in the extracts that were prepared from the stems, flowers, leaves, and roots of *E. spinosum*. In a preliminary step, (HR) LC-ESI-Orbitrap-MS and (HR) LC-ESI-Orbitrap-MS/MS analyses of *E. spinosum* extracts, namely, stems, roots, flowers, and leaves, were conducted in negative and positive ion modes. The negative ionization mode was selected based on the larger number of compounds that were detected as giving good ionization. The negative LC-MS metabolic profiles highlighted the presence of 45 metabolites, most of them identified or tentatively identified by their *m/z* values obtained through high-resolution mass spectrometry, extracted from the total ion current (TIC) profile, and implemented with the MS/MS fragmentation obtained in LC/ESI/(Orbitrap). MS/MS experiments were accomplished using a dependent data scan to submit the major ions in TIC profiles to fragmentation experiments using the MS parameters previously selected by ESI/MS and ESI-MS/MS direct introduction experiments. The registered fragmentation information was compared with the literature data of the selected compounds (Table 1). Experimental MS/MS spectra were compared with literature fragmentation patterns or those reported in a public repository of mass spectral data, such as Mass Bank [27], and associated with a comparison with a database like KNapSacK (www.knapsackfamily.com accessed on 12 July 2021). This information was coupled with a study of the genus *Echinopsis* and the chemotaxonomy of the Asteraceae family, which allowed the identification of most of the compounds, with the exception of compounds reported as unknown, as illustrated in Table 1, following a metabolomics approach, as reported in the literature for different species [17,28]. The use of pure commercial standards enabled the distinction of compounds with identical *m/z* values and MS/MS fragmentation patterns. The identification of compounds based on high-resolution mass spectrometry data, chemical formulas derived from accurate mass measurements, retention times, MS/MS results, and literature references are illustrated in Table 1. High-resolution mass values did not differ by more than 5 ppm with respect to the exact mass calculated for the same molecule (Figure 1). 

The negative ion LC–MS metabolite profiles highlighted the presence of 45 peaks corresponding to 45 compounds, 10 of which are unidentified. Interestingly, only five of the identified compounds were previously reported from *E. spinosus*, namely, campesterol (**22**), stigmasterol (**24**), apigenin-7-*β*-*O*-(4″-*O*-*trans*-*p*-coumaroyl-glucoside (**25**), cholesterol (**29**), and brassicasterol (**30**) [15,29,30,31]. Eleven compounds were previously reported in other *Echinops* species and were identified here for the first time in *E. spinosus* as quinic acid (**2**), neochlorogenic acid (3-*O*-caffeoylquinic acid) (**6**), chlorogenic acid (5-*O*-caffeoylquinic acid) (**7**), 3,5-dicaffeoylquinic acid (cynarin) (**9**), apigenin-6-*O*-arabinoside-8-*O*-galactoside (**13**), 3,4-dicaffeoylquinic acid (**14**), apigenin-6-*O*-arabinoside-8-*O*-glucoside (**15**), 3,5-dicaffeoylquinic acid (**16**), 4,5-dicaffeoylquinic acid (**17**), luteolin-7-*O*-glucoside (**18**), and rutin (**21**) [32,33,34,35,36]. However, seven compounds were previously reported in plants of the Asteraceae family, but not from the *Echinops* species, and have been identified as protocatechuic acid hexoside (**4**), dicaffeoyl altraric acid (**8**), dicaffeoyl altraric acid isomer (**12**), tricaffeoylaltraricric acid **(19)**, shimobashiraside C (**23**), trihydroxy-octadecadienoic (9,12,13-trihydroxy-10,15-octadecadienoic acid, (9,12,13,TriHODE(10,15)) acid (**28**), and trihydroxy-octadecenoic acid (9,12,13-trihydroxy-10-octadecenoic acid, 9,12,13-TriHOME(10), pinellic acid) (**33**) [32,37,38,39,40]. However, five compounds were identified for the first time in the genus *Echinops* and in the family Asteraceae. Compound (**5**) showed a molecular ion at *m/z* [M − H]^−^ 359.07479, corresponding to a molecular formula of C_18_H_15_O_8_. The fragmentation spectrum showed a main fragment ion at *m/z* 197.05 [M − H − 162]^−^ (corresponding to the loss of one unit of a hexose); a similar fragmentation was previously reported by Abdel et al. [41] and the compound was thus identified as a syringic acid glycoside. Compound (**11**) showed a molecular ion at *m/z* [M − H]^−^ 677.1714, corresponding to molecular formula C_31_H_33_O_17_. The MS/MS spectrum showed two main fragment ions at *m/z* 515.14 [M − H − 162]^−^ (corresponding to the loss of one hexose unit) and at *m/z* 353.09; through database searching, the metabolite was identified as dicaffeoylquinic acid glycoside. Compound (**20)** showed a molecular ion at *m/z* [M − H]^−^ 499.1237, corresponding to the molecular formula C_25_H_23_O_11_. In this instance, three main fragment ions were observed at *m/z* 353.09, 337.09, and 191.06, and the compound was identified as a coumaroyl-caffeoylquinic acid. Compound (**36)** showed a molecular ion at *m/z* [M − H]^−^ 579.1497, corresponding to C_30_H_27_O_12_. The fragmentation spectrum showed two main fragment ions at *m/z* 271.06 and 307.08, and the metabolite was identified as a naringenin-coumaroyl-glucopyranoside, previously reported in the genus *Crataegus* [42]. Compound (**40**) showed a molecular ion at *m/z* [M − H]^−^ 619.1443, corresponding to C_32_H_27_O_13_. In this case, the fragmentation spectrum showed a main fragment ion at *m/z* 269, and the compound was proposed to be a derivative of apigenin.

### 3.2. Quantitative Determination of E. spinosus Major Phenolic Compounds Using HPLC-DAD

Quantitative investigation of *E. spinosus* extracts was performed on targeted phenolic compounds, especially the caffeoylquinic acid derivatives, and the results are reported as mg/g dried residue (Table 2). This is the first quantitative metabolite data report for *E. spinosus*. Among the monocaffeoyl quinic derivatives, the most abundant is chlorogenic acid (5-*O*-caffeoylquinic acid), which showed the highest content in the leaves estimated at 74.30 ± 1.92 mg/g dried residue, in addition to the highest content of neochlorogenic acid (3-*O*-caffeoylquinic acid, 4.77 ± 0.57 mg/g dried residue). Among the dicaffeoyl quinic acids, the dominant compounds are the isomers 3,5-dicaffeoylquinic (3,5-diCQ) and 4,5-dicaffeoylquinic (4,5-diCQ) acids, which together represented 50 to 72% of all the hydroxy cinnamic derivatives in the extracts of *E. spinosus*. It is interesting to note that the quantitative relationships between these two acids change according to the botanical part extracted. In the extracts of the stems, roots, and flowers, the 3,5-diCQ isomer is always similar or in a slightly greater concentration than the 4,5-diCQ isomer, whereas in the leaves the concentration of 4,5-diCQ acid is almost twice that of 3,5-diCQ acid (104.85 ± 9.02 vs. 68.79 ± 0.22 mg/g dried residue, respectively). Flavonoids were found to be most expressed in the flowers (21.32 ± 0.08 mg/g dried residue), followed by the leaves (8.55 ± 0.07 mg/g dried residue) and roots (3.98 ± 0.21 mg/g dried residue), whereas traces were detected in the stems. The basic aglycones were typically apigenin, luteolin, naringenin, hesperetin, hispidulin, and quercetin. The dominant compounds in the flower and leaf extracts were hesperidin (hesperetin-7-rutinoside) and naringenin-coumaroyl-glucoside, the roots contained hispidulin, estimated by 3.98 ± 0.21 mg/g dried residue, and in contrast, traces were detected in the stems. Among the hydroxybenzoic acid derivatives it was possible to identify a protocatechuic acid hexoside and shimobashiraside C. The former was particularly concentrated in the leaves and roots, with values estimated as 2.66 ± 0.10 and 2.90 ± 0.08 mg/g dried residue, respectively, whereas the latter existed more in the leaves and stems, with values equal 2.86 ± 0.09 and 1.15 ± 0.04 mg/g dried residue, respectively.

A comparison of the metabolite profiles for the four plant parts revealed that the compounds occurring in all plant parts were protocatechuic acid hexoside and the caffeoylquinic acid derivatives, mainly chlorogenic acid, 3,5-dicaffeoylquinic acid, and 4,5-dicaffeoylquinic acid. These are 3,4-dihydroxycinnamic acid (HCA) derivatives, and their abundance is likely very important biologically. HCAs are considered significant from a nutritional point of view, owing to their antioxidant activities as catechols in addition to their protective effects against cancer and heart disease [45,46].

### 3.3. Discrimination of the Different E. spinosus Organs Using Multivariate Data Analysis

**Figure 2:**
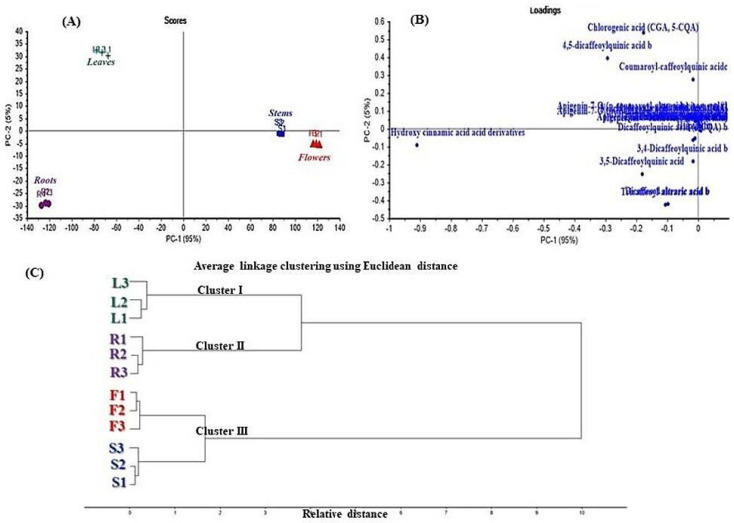
LC-based chemometrics analysis of different *Echinopsis spinosus* L. organs. (**A**) Score plot; (**B**) loading plot; (**C**) HCA.

Chemometric analysis was performed by adopting unsupervised pattern recognition represented by principal component analysis (PCA) and hierarchical cluster analysis (HCA) based upon the qualitative and quantitative LC data (Figure 2). Principal component analysis (PCA) was primarily established to classify data and thus correlate the tested samples with the utilized variables [19].

The score plot illustrated in Figure 2A effectively discriminates different *E. spinosus* organs into three distinct clusters, wherein the stems and flowers are allocated in the same cluster, reflecting their resemblance in their secondary metabolites, which undoubtedly influence their bioactivity, in contrast to the leaves and roots, which appeared in distant clusters. The PCA score plot for principal components (PCs), which were PC1 versus PC2, accounted for 95% and 5% of the total variance, respectively. Both PCs significantly discriminated between leaves that in the upper left quadrant, showing negative values for PC1 and positive values for PC2, and stems and roots in the lower right quadrant, displaying positive values for PC1 and negative values for PC2. Meanwhile, PC1 effectively distinguished between roots in the lower left quadrant, displaying negative values, and between stems and flowers. Furthermore, PC2 differentiated between leaves (positive values) and roots (negative values). By comprehensive interpretation of the loading plot (Figure 2B), it can be concluded that hydroxy cinnamic acid derivatives, chlorogenic acid, 4,5-dicaffeoylquinic acid, and coumaroyl-caffeoylquinic acid represent the main discriminatory signals among the four organs. Furthermore, HCA clustering was done with the aim of ascertaining the results obtained from PCA, wherein samples were clustered into three clusters, as illustrated in the HCA dendrogram (Figure 2C). Both flowers and stems were clustered together (cluster III) with short distance between them, compared to the roots and leaves, which formed two clusters (clusters I and II). Thus, the HCA dendrogram further confirmed the results displayed by PCA, revealing the similarity between the stems and flowers, as evidenced by their clustering in one cluster.

### 3.4. Determination of the Antioxidant Activity of E. spinosus Extracts

The antioxidant activity was measured in vitro by employing the ferric reducing/antioxidant power (FRAP), cupric reducing antioxidant activity (CUPRAC), and free radical-scavenging activity (DPPH^●^ and ABTS^●+^) assays for the *E. spinosus* extracts. The results of the performed assays, expressed based on g of dry residue, show approximately the same trends among the four examined extracts (Table 3). *E. spinosus* root extract exhibited the highest antioxidant activity, as evidenced by 3.26 and 1.61 mmol Fe^2+^/g dried residue for CUPRAC and FRAP assays, respectively, as well as great free radical-scavenging activity potential, as estimated by values of 0.53 and 0.82 mmol TEAC/g dried residue for DPPH^●^ and ABTS^●+^, respectively. In contrast, the stem extract exerted the least antioxidant activity, with a total antioxidant activity of 0.89 and 2.03 mmol Fe^2+^/g dried residue for FRAP and CUPRAC, respectively, and the free radical-scavenging activity was 0.37 and 0.47 mmol TEAC/g dried residue for DPPH^●^ and ABTS^●+^, respectively. Flower and leaf extracts showed similar antioxidant values, in between those of the stems and roots, but closer to the stem values.

### 3.5. Determination of the Total Phenolic and Flavonoid Contents of E. spinosus Extracts

The total polyphenol (TP) and the total flavonoid (TF) contents were determined that strongly correlated with the results of the antioxidant activity. The roots showed the highest phenolic and flavonoid contents, estimated by 125.16 mg GAE/g dried residue, 140.12 mg CE/g dried residue, and 25.40 mg QE/g dried residue for TP, TF II, and TF I, respectively. Meanwhile, the stem extract revealed the lowest in TP and TF II, estimated by 83.60 mg GAE/g dried residue and 105.41 mg CE/g dried residue, respectively. Furthermore, the flowers showed the lowest level of flavonoids, with TF I equal to 9.22 mg QE/g dried residue. Good positive correlations were observed between the TP content measured by the Folin–Ciocalteu method and the four antioxidant activity values and was statistically significant (*p* ≤ 0.05) with DPPH**^●^** (*r* = 0.9577). In addition, a good correlation was observed between all the antioxidant activity assays and the TF content and was statistically significant (*p* ≤ 0.05) for TFI/FRAP (*r* = 0.9464). Finally, it was observed that the TP content was significantly correlated with the total TF II (*r* = 0.9751). Direct comparison of the obtained results with the literature on *E. spinosus*, as well as other *Echinops* species, is not straightforward due to the different plant parts investigated and the extracting solvents used [47]. *E. spinosus* roots collected from Tunisia extracted with different solvents (ethanol, chloroform, hexane, and ethyl acetate) were examined for their investigated TP and TF, as well as DPPH^•^ antioxidant activity. It was shown that the TP, TF, and antioxidant activity highly relied upon the solvent polarity, and as expected, ethanol extracts were rich in phenolics compared to non-polar extracts. Moreover, the ethanol and methanol extracts of the leaves and seeds of *E. ritro* L. and *E. tournefortii* Ledeb were evaluated for their TP content and antioxidant activity. For both *Echinops* species, the methanol extracts showed the highest TP content and antioxidant activity measured by the DPPH**^●^** assay. These findings support the choice in this investigation to extract *E. spinosus* with a hydro-alcohol mixture. The ethanol extract of *E. spinosus* roots showed lower values for TP, TF, and DPPH**^●^** antioxidant activity than previously demonstrated by Khedher et al. [47]. Furthermore, a previous investigation of the hydroalcoholic extracts (MeOH:H_2_O, 70:30 *v*/*v*) of the aerial and root parts of *E. spinosus* revealed the presence of 36.1 mg EAG/100 g dry matter and 13.37 mg EC/100 g dry matter of TP and TF, respectively, in the aerial parts, and the roots contained 16.1 mg EAG/100 g dry matter of TP and 4.78 mg EC/100 g dry matter of TF [48]. A study performed on 70% ethanol extracts of *E. spinosus* above-ground parts growing in Egypt revealed lower values of TP, TF, and antioxidant activity [49], which may have been attributed to the difference in geographical region, the solvent used, and the extraction procedure, whereas in the current study an effective ultrasound-assisted extraction was performed twice.

### 3.6. Computer-Aided Drug Design Studies

#### 3.6.1. Molecular Docking

**Figure 3:**
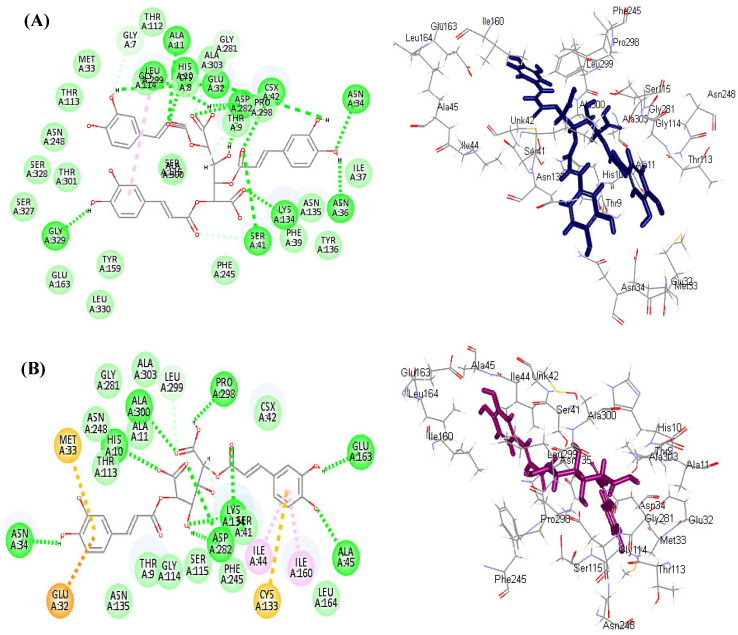
2D and 3D binding mode of tricaffeoyl-altraric acid (**A**) and dicaffeoyl-altraric acid (**B**) identified in different *Echinopsis spinosus* L. organs in the binding site of NADPH oxidase (NO).

In silico molecular modeling for the major polyphenolic compounds identified from different organs of *E. spinosus* methanol extract was done within the active sites of NADPH oxidase (NO) and myeloperoxidase (MP), which are responsible for the generation of reactive oxygen species (ROS), to estimate their enzymatic inhibitory potential. The results illustrated in Table 4 reveal that most of the tested compounds showed inhibition to both enzymes with varying degrees; however, tricaffeoyl-altraric acid followed by dicaffeoyl-altraric acid exhibited the best fit within the active site of NADPH oxidase (NO) and myeloperoxidase (MP), displaying binding energies (∆G) of −93.93 and −81.80 Kcal/mol, respectively, for NADPH oxidase (NO), and ∆G) of −75.35 and −60.52 Kcal/mol, respectively, for myeloperoxidase (MP). This firm fitting within the active site of the enzymes can be explained by the virtue of the formation of many bonds. Concerning NADPH oxidase (NO), tricaffeoyl-altraric acid formed 13 conventional H-bonds with Asp282, Lys134, Ser41, Asn34, Asn36, Glu32, Ala11, Csx42, Gly329, and Leu299; one π-alkyl bond with Glu114; and one C-H bond with Gly7 existing at the active site, together with many Van der Waals interactions (Figure 3A). Meanwhile, dicaffeoyl-altraric formed nine conventional H-bonds with Asp282, Glu163, Ala45, Lys134, His 10, ALa300, and Pro 298; two π-alkyl bonds with Ile44 and Ile160; one π-anion bond with Glu32; two π-sulfur bonds with Met33 and Cys133; one C-H bond with Leu299; and many Van der Waals interactions (Figure 3B).

**Figure 4:**
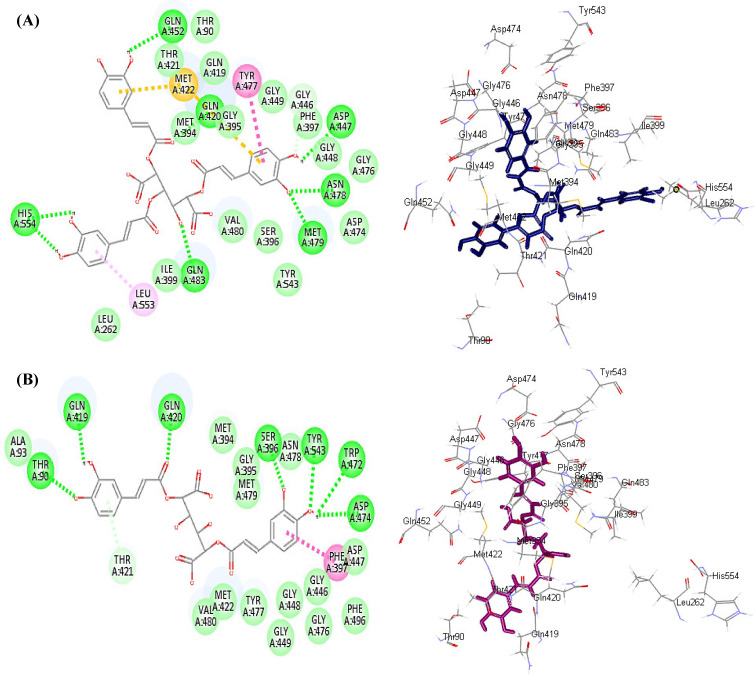
2D and 3D binding mode of tricaffeoyl-altraric acid (**A**) and dricaffeoyl-altraric acid (**B**) identified in different *Echinopsis spinosus* L. organs in the binding site of myeloperoxidase (MP).

Regarding myeloperoxidase (MP), tricaffeoyl-altraric acid formed eight H-bonds with His554, Gln483, Met479, Asp447, Aasn478, Gln420, and Gln452; one π-π T-shaped bond with Tyr477; a π-alkyl bond with Leu535; two π-sulfur bonds with Met33; and one C-H bond with Gly466, together with the formation of multiple Van der Waals interactions (Figure 4A). However, dicaffeoyl-altraric formed seven H-bonds with Asp474, Thr90, Gln419, Gln420, Ser396, Tyr543, and Trp472; one π-π T-shaped bond with Phe397; one C-H bond with Thr421; and many Van der Waals interactions (Figure 4B). The results of molecular docking further support the obtained in vitro results, wherein both tricaffeoyl-altraric acid and dicaffeoyl-altraric acid existed in a higher concentration in the roots, estimated at 38.97 and 46.01 mg/g dried residue, respectively, compared to other organs and showed the highest antioxidant capacity, as revealed in all the performed assays.

#### 3.6.2. ADME/TOPAKT Prediction

**Figure 5:**
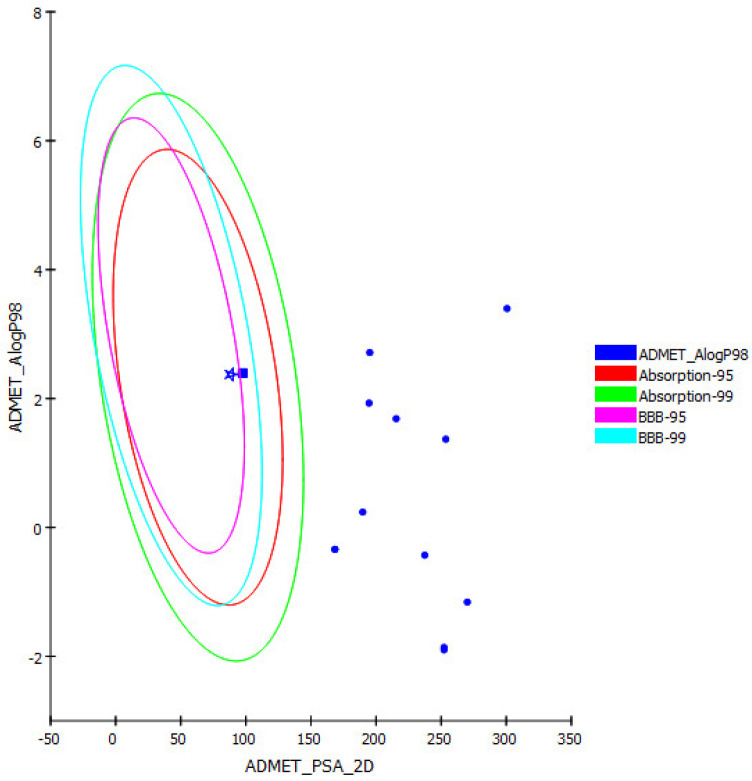
ADMET plot of *E. spinosus* major phenolic compounds displaying 95% and 99% confidence limit ellipses with respect to the human intestinal absorption and the blood–brain barrier (BBB) models; hispidulin (filled square); naringenin (filled star).

*E. spinosus* major phenolic compounds were subjected to ADME/TOPAKT evaluations to assess their pharmacokinetic, pharmacodynamic, and toxicity properties in silico using Discovery Studio 4.5 (Accelrys Inc., San Diego, CA, USA). The results presented in Table 5 show that all the examined compounds displayed low human intestinal absorption except for hispidulin and naringenin, which showed good human intestinal absorption and hence were allocated inside the 95% absorption ellipse, as revealed in the ADMET plot (Figure 5). Besides, most of the tested compounds showed low to very low solubility, except for naringenin, hispidulin, luteolin-7-*O*-glucoside, and coumaroyl-caffeoylquinic acid, which displayed good solubility. Meanwhile, chlorogenic acid and neochlorogenic acid showed optimal solubility. With respect to BBB, all the compounds showed undefined BBB, taking value 4 and appearing outside the 99% confidence eclipse of BBB, whereas hispidulin and naringenin revealed low penetration via BBB and thus appeared within the 99% confidence eclipse of BBB in the ADMET plot. Furthermore, all the tested compounds showed less than 90% plasma protein binding (PPB). None of the tested compounds inhibited CPY2D6 except for apigenin 6-arabinoside-8-glucoside and naringenin, which displayed certain inhibitory potential versus CPY2D6. Regarding hepatotoxicity, apigenin 6-arabinoside-8-glucoside, hesperidin, hispidulin, luteolin-7-*O*-glucoside, naringenin, and rutin showed a certain degree of toxicity to hepatocytes, whereas other compounds revealed no hepatotoxicity.

Concerning the TOPKAT evaluation, all the tested compounds showed a non-mutagenic effect, as evidenced by the Ames prediction. They also exerted no carcinogenic effect towards either male or female rat NTP, except for apigenin-6-arabinoside-8-galactoside, hispidulin, and naringenin, which showed a certain degree of carcinogenic effect versus male rat NTP only. In addition, the tested *E. spinosus* major phenolic compounds showed rat oral LD50 values in the range of 0.47 and 11.14 g/kg body wt. Meanwhile, they displayed rat inhalational LD_50_ values ranging between 1.79 and 3435.69 mg/m^3^/h with LOAEL (lowest-observed-adverse-effect level) values between 0.01 and 0.27/kg body wt. None of the tested compounds revealed irritation to the skin. However, most of the compounds showed mild to moderate eye irritation, except for naringenin-coumaroyl-glucoside, which exhibited no irritation, in contrast to apigenin 6-arabinoside-8-glucoside, apigenin-6-arabinoside-8-galactoside, dicaffeoyl altraric acid, and tricaffeoyl-altraric acid, which revealed severe ocular irritancy. Moreover, naringenin was the only compound among all the tested *E. spinosus* major phenolic compounds that revealed aerobic non-biodegradable behavior (Table 6).

From the ADME/TOPAKT analyses, it can be concluded that most of the compounds revealed acceptable toxicity properties. However, the pharmacokinetics and pharmacodynamics require some treatment to be suitable for incorporation into pharmaceutical dosage forms. It is worth highlighting that tricaffeoyl-altraric acid and dicaffeoyl-altraric acid, which exhibited the best binding capacity from the molecular docking study and concomitantly promising antioxidant capacity, also revealed reasonable pharmacokinetics and pharmacodynamics, with a significantly safe profile.

## 4. Conclusions

A comparative study on the qualitative and quantitative analysis of the polyphenolics in aqueous methanol extracts of the leaves, stems, flowers, and roots of *E. spinosus* is described herein for the first time. *Echinops* extracts constitute rich sources of polyphenols and thus could be used as powerful natural antioxidants, particularly the root extracts. The methanol extract of the roots demonstrated the highest reducing activity, whereas less activity was observed for the methanol extract of the stems. These results highlight the good correlation between the antioxidant activity and the phenolic content, with the highest value observed for the root extract. Additionally, molecular docking revealed that most of the tested compounds showed inhibition to both enzymes with varying degrees; however, tricaffeoyl-altraric acid, followed by dicaffeoyl-altraric acid, exhibited the best fit within the active site of NADPH oxidase (NO) and myeloperoxidase (MP). The results of molecular docking further ascertain the obtained in vitro results, with both tricaffeoyl-altraric acid and dicaffeoyl-altraric acid existing in the highest concentrations in the roots compared to other organs, and showed the highest antioxidant capacity, as revealed in all the performed assays. From the ADME/TOPAKT analyses, it can be concluded that most of the compounds revealed acceptable toxicity properties. However, the pharmacokinetics and pharmacodynamics require some treatment to be suitable for incorporation into pharmaceutical dosage forms. It is worth highlighting that tricaffeoyl-altraric acid and dicaffeoyl-altraric acid, which exhibited the best binding capacity from the molecular docking study and concomitantly promising antioxidant capacity, also revealed reasonable pharmacokinetics and pharmacodynamics, with a significantly safe profile. However, assessment of the antioxidant and toxicity profile of *Echinops* extracts in vivo is an important next step so that their safety limits as a dietary antioxidant source for human health can be established, recognizing that the roots are already used as a spice in Morocco and Cameroon.

## Figures and Tables

**Table 1:** Qualitative identification of major compounds in *E. spinosus* extracts (S, stems; R, roots; F, flowers; L, leaves; X, present; -, absent) by LC-ESI-Orbitrap-MS and LC-ESI-Orbitrap-MS/MS analysis.

Peak No.	R_t_	[M − H]^−^	Molecular Formula	Δ Ppm	MS/MS	Identity	S	R	F	L	References
1	1.87	377.0855	C_18_H_17_O_9_	−3.258	341.11	Caffeic acid derivative	X	X	-	-	[27]
2	1.91	191.0563	C_7_H_11_O_6_	6.833	85.03/93.04/127.04/173.05	Quinic acid	X	-	-	X	[34,43]
3	5.83	329.0871	C_14_H_17_O_9_	1.281	167.04	Unknown	-	-	-	X	-
4	6.06	315.0715	C_13_H_15_O_9_	1.592	153.02	Protocatechuic acid hexoside	X	X	X	X	[32]
5	6.33	359.0747	C_18_H_15_O_8_	−3.77	197.05	Syringic acid glycoside	-	-	X	-	[44]
6	7.24	353.0872	C_16_H_17_O_9_	1.449	179.03/191.06	Neochlorogenic acid (3-CQA)	-	X	X	X	[34]
7	8.46	353.0872	C_16_H_17_O_9_	1.449	179.03/191.06	Chlorogenic acid (5-CQA)	X	X	X	X	[34]
8	9.65	533.0929	C_24_H_21_O_14_	0.597	371.06/209.03	Dicaffeoyl altraric acid	X	X	-	-	[39]
9	9.96	515.1187	C_25_H_23_O_12_	0.713	353.09	3,5-Dicaffeoylquinic acid	-	X	-	X	[36]
10	10.93	565.1919	C_27_H_33_O_13_	0.695	327.12/339.12	Unknown	X	-	-	-	
11	11.16	677.1714	C_31_H_33_O_17_	0.242	515.14/353.09	Dicaffeoylquinic acid glycoside	X	-		-	[27]
12	11.42	533.09271	C_24_H_21_O_14_	0.241	371.06	Dicaffeoyl altraric isomer acid	-	X	-	-	[39]
13	11.78	563.1417	C_26_H_27_O_14_	3.860	-	Apigenin-6-*O*-arabinoside-8-*O*-galactoside	-	-	X	-	[33]
14	12.43	515.1182	C_25_H_23_O_12_	−0.587	353.08	3,4-Dicaffeoylquinic acid	X	X	X	X	[36]
15	12.47	563.1417	C_26_H_27_O_14_	3.860	-	Apigenin-6-*O*-arabinoside-8-*O*-glucoside	-	-	X	-	[33]
16	12.89	515.1182	C_25_H_23_O_12_	1.781	353.08	3,5-Dicaffeoylquinic acid	X	X	X	X	[36]
17	13.24	515.1187	C_25_H_23_O_12_	0.248	353.08	4,5-Dicaffeoylquinic acid	X	X	X	X	[36]
18	13.40	447.0932	C_21_H_19_O_11_	1.101	-	Luteolin-7-*O*-glucoside	-	-	X	-	[33]
19	14.09	695.1241	C_33_H_27_O_17_	0.337	533.09/371.06	Tricaffeoylaltraricric acid	X	X	-	-	[39]
20	14.20	499.1237	C_25_ H_23_ O_11_	0.585	353.09/337.09/191.06	3-*p*-(*E*)-Coumaroyl-5-(*E*)-caffeoylquinic acid	X	-	-	X	[34]
21	14.34	609.1602	C_27_H_29_O_16_	1.010	301	Rutin	-	-	X	-	[33]
22	14.57	399.3621	C_28_H_47_O	−0.107	152.01/153.02/315.07	Campesterol	-	-	X	-	[30,31]
23	15.55	435.0920	C_20_H_19_O_11_	−0.340	297.06/315.07	Shimobashiraside C	X	-	-	X	[38,40]
24	15.95	411.3617	C_29_H_47_O	−0954	315.07	Stigmasterol	-	-	X	-	[30,31]
25	16.07	577.1341	C_30_H_25_O_12_	0.060	269.05	Apigenin-7-*β*-d-*O*-(*p*-coumaroyl)-glucoside isomer	-	-	X	X	[15,29]
26	16.17	357.1914	C_18_H_29_O_7_	1.765	198.02	Unknown	-	-	-	X	-
27	16.42	582.2596	C_21_H_44_O_17_N	−1.360	462.20	Unknown	-	-	X	-	-
28	16.84	327.2174	C_18_H_31_O_5_	2.290	171.10/211.13/229.14/291.20	9,12,13-TriHODE (10,15)	X	X		X	[32]
29	16.88	385.3458	C_27_H_45_O	−1.610	-	Cholesterol	-	-	X	-	[30,31]
30	17.09	397.3451	C_28_H_45_O	−3.501	-	Brassicasterol	-	-	X	-	[30,31]
31	17.16	519.1862	C_26_H_31_O_11_	0.370	213.09/475.20	Unknown	-	X	-	-	-
32	17.29	665.3169	C_34_H_49_O_13_	0.289	503.29	Unknown	X	-	-	-	-
33	17.76	329.2330	C_18_H_33_O_5_	2.276	211.13/229.14	9,12,13-TriHODE (10)	X	X	-	-	[32]
34	17.79	577.1343	C_30_H_25_O_12_	0.491	269.04/413.09/431.10	Apigenin-7-β-d-*O*-(*p*-coumaroyl)-glucoside isomer	-	-	X	X	[15,29]
35	17.90	609.1602	C_27_H_29_O_16_	2.011	-	Hesperidin	-	-	X	-	[27,33]
36	18.26	579.1497	C_30_H_27_O_12_	1.022	271.06/307.08	Naringenin-coumaroyl-glucoside	-	-	X	X	[27]
37	18.31	605.1866	C_29_ H_33_ O_14_	0.271	561.20	Unknown	-	X	-	-	-
38	18.31	299.0556	C_16_ H_11_ O_6_	1.990	-	Hispidulin	-	X	-	-	[27]
39	18.37	609.1603	C_27_H_29_O_16_	2.010	-	Luteolin-Ara-Glu or Luteolin-Glu-Ara	-	-	X	-	[27,33]
40	18.62	619.1443	C_32_H_27_O_13_	−1.040	269	Apigenin derivative	-	-	X	X	[27]
41	23.84	445.2431	C_22_H_37_O_9_	−0.178	198.01/283.86	Unknown	-	X	-	-	-
42	26.56	761.2856	C_34_H_49_O_19_	−0.808	198.00/283.86/633.24	Unknown	-	X	-	-	-
43	29.55	295.2273	C_18_H_31_O_3_	2.096	171.10/277.22	10,12-Octadecadienoic acid, 9-hydroxy-	-	X	-	-	[27]
44	33.27	513.3062	C_27_H_45_O_9_	0.781	198.01/283.86	Unknown	-	X	-	-	-
45	36.98	271.0607	C_16_H_17_O_9_	0.101	-	Naringenin	-	-	X	-	[27,33]

**Table 2:** Concentration of targeted polar compounds (mg/g dried residue) in *E. spinosus* L. extracts (mean ± SD, *n* = 3).

Compound	Identification ^a^	Extract (mg/g Dried Residue)			
		Stems	Roots	Flowers	Leaves
Hydroxy cinnamic acid derivatives		135.28 ± 1.42	330.20 ± 0.98	105.95 ± 0.41	284.86 ± 2.9
Neochlorogenic acid (NCGA, 3-CQA)	Rt, UV-Vis, MS	1.54 ± 0.22	2.19 ± 0.14	1.51 ± 0.07	4.77 ± 0.57
Chlorogenic acid (CGA, 5-CQA)	Rt, UV-Vis, MS	22.56 ± 0.25	46.01 ± 0.56	17.18 ± 0.34	74.30 ± 1.92
Dicaffeoyl altraric acid ^b^	UV-Vis, MS	4.14 ± 4.24	35.95 ±0.02	1.10 ± 0.02	3.83 ± 0.12
Dicaffeoylquinic acid (diCQA) ^b^	UV-Vis, MS	2.71 ± 0.13	6.68 ± 0.19	0.29 ± 0.01	1.76 ± 0.08
3,4-Dicaffeoylquinic acid ^b^	UV-Vis, MS	4.58 ± 0.01	16.18 ± 0.72	10.34 ± 0.01	3.69 ± 0.1
3,5-Dicaffeoylquinic acid	Rt, UV-Vis, MS	44.13 ± 2.69	93.11± 3.13	45.50 ± 1.25	68.79 ± 0.22
4,5-Dicaffeoylquinic acid ^b^	UV-Vis, MS	40.57 ± 1.15	91.12 ± 1.34	29.26 ± 0.54	104.85 ± 9.02
Tricaffeoyl-altraric acid ^b^	UV-Vis, MS	6.84 ± 0.32	38.97 ± 1.32	0.76 ± 0.07	5.94 ± 0.5
Coumaroyl-caffeoylquinic acid ^c^	UV-Vis, MS	8.22 ± 0.64	tr	tr	17.44 ± 0.34
Flavonoids		tr	3.98 ± 0.21	21.32 ± 0.08	8.55 ± 0.07
Apigenin-6-arabinoside-8-galactoside^d^	UV-Vis, MS	nd	nd	1.87 ± 0.01	nd
Apigenin 6-arabinoside-8-glucoside ^d^	UV-Vis, MS	nd	nd	0.92 ± 0.01	nd
Luteolin-7-*O*-glucoside	Rt, UV-Vis, MS	tr	tr	1.91 ± 0.01	tr
Quercetin-3-rutinoside (rutin)	Rt, UV-Vis, MS	nd	nd	1.65 ± 0.23	nd
Apigenin-7-*O*-(*p*-coumaroyl-glucoside) isomer ^d^	UV-Vis, MS	tr	tr	1.32 ± 0.01	3.68 ± 0.05
Apigenin-7-*O*-(*p*-coumaroyl-glucoside) isomer ^d^	UV-Vis, MS	nd	nd	1.81± 0.01	2.07 ± 0.02
Hesperetin-7-rutinoside (Hesperidin)	Rt, UV-Vis, MS	tr	tr	3.00 ± 0.07	tr
Naringenin-coumaroyl-glucoside ^f^	UV-Vis, MS	tr	tr	4.01 ± 0.23	1.36 ± 0.02
Hispidulin	Rt, UV-Vis, MS	tr	3.98 ± 0.21	nd	nd
Luteolin ara-glu/glu-ara ^e^	UV-Vis, MS	nd	nd	0.94 ± 0.03	nd
Apigenin glucosidated ^d^	UV-Vis, MS	nd	nd	3.02 ± 0.02	1.44 ± 0.06
Naringenin	Rt, UV-Vis, MS	nd	nd	0.88 ± 0.03	nd
Hydroxybenzoic acid derivatives		2.46 ± 0.001	2.90 ± 0.08	0.93 ± 0.02	5.52 ± 0.01
Protocatecuic acid hexoside ^g^	UV-Vis, MS	1.31 ± 0.04	2.90 ± 0.08	0.93 ± 0.02	2.66 ± 0.1
Shimobashiraside C ^g^	UV-Vis, MS	1.15 ± 0.04	tr	tr	2.86 ± 0.09

^a^: Rt, comparison with retention time of pure standard; UV-Vis, comparison with UV-VIS spectra of pure compound or similar pure standards; MS, MS/MS spectra fragmentation patterns reported in the literature as described in Table 1. ^b^: Determined with the calibration curve of 3,5-dicaffeoylquinic acid. ^c^: Determined with the calibration curve of chlorogenic acid. ^d^: Determined with the calibration curve of apigenin-7-*O*-glucoside. ^e^: Determined with the calibration curve of luteolin-7-*O*-glucoside. ^f^: Determined with the naringenin calibration curve. ^g^: Determined with the protocatechuic acid calibration curve. The results are reported as mean value ± standard deviation (*n* = 3); nd: not detected (<LOD); tr, traces (<LOQ).

**Table 3:** Antioxidant capacities, total phenols, and total flavonoids of different *E. spinosus* extracts (per g of extract dry residue).

Samples	CUPRAC ^a^	FRAP ^a^	DPPH^● b^	ABTS^●+ b^	TP ^c^	TF I ^d^	TF II ^e^
Stems	2.03 ± 0.09 ^a^	0.89 ± 0.04 ^a^	0.37 ± 0.02 ^a^	0.47 ± 0.03 ^ac^	83.60 ± 3.64 ^a^	10.64 ± 1.63 ^a^	105.41 ± 2.79 ^a^
Roots	3.26 ± 0.19 ^b^	1.61 ± 0.14 ^b^	0.53 ± 0.01 ^b^	0.82 ± 0.04 ^b^	125.16 ± 9.48 ^b^	25.40 ± 1.76 ^b^	140.12 ± 1.48 ^b^
Flowers	2.50 ± 0.12 ^c^	1.06 ± 0.08 ^c^	0.42 ± 0.03 ^ac^	0.52 ± 0.02 ^ac^	97.59 ± 4.25 ^c^	9.22 ± 1.31 ^a^	124.71 ± 7.95 ^c^
Leaves	2.37 ± 0.30 ^ac^	1.02 ± 010 ^ac^	0.47 ± 0.02 ^c^	0.46 ± 0.02 ^a^	121.50 ± 11.25 ^b^	13.15 ± 0.73 ^c^	138.10 ± 1.94 ^d^

^a^ FRAP (ferric ion-reducing antioxidant power) and CUPRAC (cupric ion-reducing antioxidant capacity) values are expressed as Fe^2+^ millimolar concentration, obtained from a FeSO_4_ solution with an antioxidant capacity equivalent to that of the dilution of the dry extract residue; mmol Fe^2+^/g dried residue. ^b^ DPPH^●^ (1.1-diphenyl-2-picrylhydrazylradical) and ABTS^●+^ (2.2′-azino-bis(3-ethylbenzothiazoline-6-sulfonate radical cation) values are expressed as TEAC millimolar concentration, obtained from a Trolox solution with an antiradical capacity equivalent to that of the dilution of the dry extract residue; mmol TEAC/g dried residue. ^c^ GAE: gallic acid equivalent; mg GAE/g dried residue. ^d^ QE: quercetin equivalent; mg QE/g dried residue. ^e^ CE: catechin equivalent; mg CE/g dried residue. Results are reported as the mean value ± standard deviation (*n* = 3). Means in the same column that do not share a letter are significantly different (*p* ≤ 0.05).

**Table 4:** Binding energies (kcal/mol) of the major polar compounds in the *E. spinosus* L. extracts within the active sites of NADPH oxidase (NO) and myeloperoxidase (MP).

Compounds	NADPH Oxidase (NO)	Number of Formed Hydrogen Bonds with the Amino Acid Residues	Myeloperoxidase (MP)	Number of Formed Hydrogen Bonds with the Amino Acid Residues
Apigenin 6-arabinoside-8-glucoside	11.64	7; Asp282, Glu163, Ala45, Lys134, Csx 42	−1.44	4; Met422, Gln420, Gly493
Apigenin-6-arabinoside-8-galactoside	−5.06	6; Asp282, Glu163, Lys134, Ser 115	14.14	5; Gly476, Asp447, Met479, Gln452
Chlorogenic acid	−40.01	7; Lys134, Ser115, Asn34, Asn36, Tyr 136	−36.55	6; Asp447, Asp474, Gly476, Gln420, Met422
Coumaroyl-caffeoylquinic acid	−60.80	5; Asp282, Lys134, Csx42, His 10, Ala11	−44.42	6; ; Asp447, Asp474, Met479. Gln482, Lys487, Glu484
Dicaffeoyl altraric acid	−81.8	9; Asp282, Glu163, Ala45, Lys134, His 10, ALa300, Pro 298	−60.52	7; Asp474, Thr90, Gln419, Gln420, Ser396, Tyr543, Trp472
Dicaffeoylquinic acid	−58.16	5; Asp282, Glu32, Lys134, Ile 160, Csx42	−49.41	4; ; Asp447, Asp474, Ser396, Gln19, Gln420
Hesperidin	−11.43	5; Lys134, Asn135, Thr9, Thr112, Ala11	−4.23	2; Gln420, Met479
Hispidulin	−37.67	2; Asp282	−30.34	3; Asp474, Gln420, Gly476
Luteolin-7-*O*-glucoside	−33.70	7; Asp282, Lys134, Pro 298, Csx42, ALa300, Glu32	−15.23	5; Asp447, Asp474, Gln420, Gly476, Tyr543
Naringenin-coumaroyl-glucosid	−38.22	6; Ala45, Ser41, Lys134, Asn34, Asp282	−23.75	2; Asp474, Tyr543
Naringenin	−36.05	3; Asp282, Glu32	−30.90	3; Asp447, Asp474, Gly476
Neochlorogenic acid	−42.63	6; Asp282, Lys134, Pro 298, ALa300, Glu32	−37.49	5; Asp447, Asp474, Gln420, Gln483, Ser396
Rutin	−12.13	8; Asp282, Lys134, Pro 298, Ser41, Asn34, Asn36, Met33	2.41	3; Asp447, Thr421, Gln419
Tricaffeoyl-altraric acid	−93.93	13; Asp282, Lys134, Ser41, Asn34, Asn36, Glu32, Ala11, Csx42, Gly329, Leu299	−75.35	8; His554, Gln483, Met479, Asp447, Aasn478, Gln420, Gln452

Positive values indicate unfavorable interaction.

**Table 5:** ADMET (absorption, distribution, metabolism, excretion, and toxicity) properties of *E. spinosus* major phenolic compounds.

Compounds	Absorption Level	Solubility Level	BBB Level	PPB Level	CPY2D6	Hepatotoxic	PSA-2D	Alog p98
Apigenin 6-arabinoside-8-glucoside	3	2	4	False	Inh.	Toxic	−1.90	252.25
Apigenin-6-arabinoside-8-galactoside	3	2	4	False	NI	NT	−1.86	252.25
Chlorogenic acid	3	4	4	False	NI	NT	−0.34	168.42
Coumaroyl-caffeoylquinic acid	3	3	4	False	NI	NT	1.93	194.66
Dicaffeoyl altraric acid	3	2	4	False	NI	NT	1.37	253.59
Dicaffeoylquinic acid	3	2	4	False	NI	NT	1.69	215.47
Hesperidin	3	2	4	False	NI	Toxic	−0.43	237.41
Hispidulin	0	3	3	False	NI	Toxic	2.39	97.61
Luteolin-7-*O*-glucoside	3	3	4	False	NI	Toxic	0.24	189.80
Naringenin-coumaroyl-glucosid	3	2	4	False	NI	NT	2.71	195.21
Naringenin	0	3	3	False	Inh.	Toxic	2.37	88.68
Neochlorogenic acid	3	4	4	False	NI	NT	−0.34	168.42
Rutin	3	1	4	False	NI	Toxic	−1.16	270.11
Tricaffeoyl-altraric acid	3	1	4	False	NI	NT	3.40	300.63

0, 1, 2, and 3 indicate good, moderate, low, and very low absorption, respectively; 0, 1, 2, 3, 4, and 5 indicate extremely low, very low but possible, low, good, optimal, and too soluble, respectively; 0, 1, 2, 3, and 4 denote very high, high, medium, low, and undefined penetration via BBB, respectively. PBB, plasma protein binding; false = less than 90%, true = more than 90%; NI: non-inhibitor; Inh.; inhibitor; NT: non-toxic.

**Table 6:** TOPKAT prediction of *E. spinosus* major phenolic compounds.

Compounds	Ames Prediction	Rat Oral LD_50_	Rat Inhalational LD_50_	Rat Chronic LOAEL	Skin Irritancy	Ocular Irritancy	Rat Female NTP	Rat Male NTP	Aerobic Biodegradability
Apigenin 6-arabinoside-8-glucoside	Non-mutagen	3.88	10.20	0.12	None	Severe	Non-carcinogen	Non-carcinogen	Degradable
Apigenin-6-arabinoside-8-galactoside	Non-mutagen	1.96	7.20	0.06	None	Severe	Non-carcinogen	Carcinogen	Degradable
Chlorogenic acid	Non-mutagen	1.97	93.17	0.03	None	Moderate	Non-carcinogen	Non-carcinogen	Degradable
Coumaroyl-caffeoylquinic acid	Non-mutagen	1.70	33.23	0.02	None	Moderate	Non-carcinogen	Non-carcinogen	Degradable
Dicaffeoyl altraric acid	Non-mutagen	7.98	9.59	0.27	None	Severe	Non-carcinogen	Non-carcinogen	Degradable
Dicaffeoylquinic acid	Non-mutagen	2.06	19.41	0.02	None	Moderate	Non-carcinogen	Non-carcinogen	Degradable
Hesperidin	Non-mutagen	2.89	39.63	0.05	None	Mild	Non-carcinogen	Non-carcinogen	Degradable
Hispidulin	Non-mutagen	0.47	3646.74	0.06	None	Moderate	Non-carcinogen	Carcinogen	Non-degradable
Luteolin-7-*O*-glucoside	Non-mutagen	1.36	100.48	0.03	None	Moderate	Non-carcinogen	Non-carcinogen	Degradable
Naringenin-coumaroyl-glucosid	Non-mutagen	2.46	72.35	0.01	None	None	Non-carcinogen	Non-carcinogen	Degradable
Naringenin	Non-mutagen	1.58	3435.69	0.08	None	Mild	Non-carcinogen	Carcinogen	Non-degradable
Neochlorogenic acid	Non-mutagen	1.97	93.17	0.03	None	Moderate	Non-carcinogen	Non-carcinogen	Degradable
Rutin	Non-mutagen	2.01	14.28	0.10	None	Mild	Non-carcinogen	Carcinogen	Degradable
Tricaffeoyl-altraric acid	Non-mutagen	11.14	1.79	0.22	None	Severe	Non-carcinogen	Non-carcinogen	Degradable

Both rat chronic LOAEL and rat oral LD50 are measured in g/kg bw; meanwhile, rat inhalational LD50 is measured in mg/m^3^/h.

## Data Availability

Data are available in the manuscript.
